# Toward overcoming physical disability in spinal cord injury: a qualitative inquiry of the experiences of injured individuals and their families

**DOI:** 10.1186/s12883-019-1391-6

**Published:** 2019-07-19

**Authors:** Alireza Nikbakht-Nasrabadi, Nooredin Mohammadi, Manijeh Yazdanshenas, Maryam Shabany

**Affiliations:** 1 Brain and Spinal Cord Injury Research Center, Neuroscience Institute, Tehran University of Medical Sciences, Tehran, Iran; 2 Department of Medical Surgical Nursing and Head of the Faculty, Nursing and Midwifery School, Tehran University of Medical Sciences, Tehran, Iran; 3 Department of Critical Care Nursing, Nursing and Midwifery School, Iran University of Medical Sciences, Tehran, Iran

**Keywords:** Spinal cord injury, Family, Physical, Disability

## Abstract

**Background:**

Spinal cord injury (SCI) is a life-changing experience for the individuals with SCI and their families. This study aimed to investigate physical strategies used for overcoming physical disability in individuals with SCI.

**Methods:**

In this qualitative study, 17 SCI persons and 13 family caregivers were selected by a purposeful sampling. Settings of the study were Brain and SCI research (BASIR) center of Tehran University of Medical Sciences and Southern Social Welfare Center of Tehran and SCI Association of Tehran, Iran. Data were collected by face-to-face semi-structured interviews, which were continued until data saturation. The gathered data were concurrently analyzed by the content analysis method.

**Results:**

The data analysis revealed one main theme (towards overcoming physical disability) and three sub-themes: 1) physical rehabilitation by various methods; 2) tendency towards the use of alternative medical methods; and 3) making effort for self-reliance.

**Conclusion:**

The participants used physiotherapy and occupational therapy as an effective and essential approach offered by the healthcare team. Some individuals with SCI with help of their family had invented simple rehabilitation equipment for help to their physical rehabilitation. However, most participants had referred to different complimentary medicine specialists based on advice friends and relatives and they often had spent a lot of time and money ineffectively. Therefore, they need training and support of the healthcare team as well as social support to achieve physical independence and physical recovery. Further research is suggested to investigate the barriers to achieving physical empowerment in people with SCI in Iran.

## Background

Spinal cord injury (SCI) is a catastrophic event that may occur suddenly and unexpectedly. It can have devastating financial consequences for the affected people [1]. The prevalence of traumatic SCI in Tehran was between 1.2 and 11.4 per 10,000 from 2003 to 2008, therefore it can be considered as an important form of disability [2]. In addition, SCI may cause permanent disability and decreased life expectancy [3].

Furthermore, it is a life-changing experience not only for the person who suffers from it, but also for his/her parents, spouse, children, and siblings [4]. Functional restrictions caused by physical problems can reduce quality of life for people with SCI and their families [5, 6] . When a family member becomes affected by SCI, the rest of family members would do their best to improve the conditions. The methods of adaptation vary from family to family [7, 8].

On the other hand, restoring independent function in work and activities daily living for overcoming physical disability in individuals with SCI related to various factors such as accessibility appropriate environment, existence of relevant technologies, people’s attitude toward disability, well trained interdisciplinary staffs, and policies that help to involvement of people with a disability in all areas of life [9]. Overcoming disability due to independent function and physical recovery in people with SCI brings personal and familial happiness, freedom, self-confidence, promotion of activities of daily living, better life quality, and better communication with other people [10, 11]. Regular physical activity is beneficial for people with movement disorders, such as those with SCI, and could be effective in management of daily life activities and mental health [12].

A comprehensive understanding of people’s experiences in overcoming physical disability requires deep and extensive investigations that can be done by qualitative research [13]. Since few studies have been conducted on this subject in developing countries, this study can contribute physical strategies experienced by individuals with SCI and their families for overcoming physical disability.

## Methods

### Study design

This research was part of a grounded theory PhD dissertation investigating experiences of individuals with SCI and their families for overcoming physical disability.

### Methodology

Content analysis method was considered appropriate for this study as it helped the researchers to understand the physical strategies used for overcoming physical disability in individuals with SCI, using the statements and experiences of the participants [14, 15].

### Sampling and data collection

Participants were selected with using a purposive sampling (identification and selection of information-rich cases related to the phenomenon), [16] from three centers in Tehran: BASIR Center, Social Welfare Center and SCI association of Tehran. Seventeen people with SCI and 13 family members as caregivers were selected from November 2015 to September 2016. All of them accepted to participate in this study. The principle maximum variation was preserved during the sampling (gender, different socio-economic levels, and different clinical situation) [17].

The inclusion criteria of individuals with SCI were: 1) being 18 years old or older, 2) being literate 3) having SCI for more than 1 year, 4) having paraplegia and tetraplegia SCI diagnosed by a specialist. The exclusion criteria were: 1) having extremity fractures, 2) having head injury and big surgery (such as heart or femur surgery, 3) having SCI caused by non-trauma conditions and 4) being pregnant.

Inclusion criteria for the family caregivers were: 1) being member of a family as a sibling, parent, spouse or child of affected individuals for more than a year, 2) literate, 3) being responsible for taking care of an individual with SCI (caregiver approved by the individual with SCI), and 4) not being a member of the treatment team.

Data were collected by deep semi-structured face-to-face interviews until data saturation. Data saturation refers to the state when data review creates no new codes anymore and the extracted categories are considered coherent and logical [14]. The interviews were performed at the convenient time and place for the participants such as participants’ home, private room in research center and hospital yard. All interviews were recorded by audio recorder and were typed verbatim. The duration of each interview was from 30 to 90 min.

### Data analysis

The first author (MS) has employed the content analysis method recommended by Graneheim and Lundman (2004) [15]. In accordance with this method, a constant comparative analysis was used simultaneously with data collection and continual review of interviews and observations. The purpose of observation in this research was to examine non-verbal behaviors and individual interactions of the spinal cord injury with the family [18]. The observations were fully written immediately upon completion of it at the right time.

Each transcript of interview was read several times. Data was broken into meaningful units then the units were labeled to create categories. It needs to mention that similar labels were combined to reduce redundancy among categories. Finally, one main theme emerged [19].

Field notes from observation were entered into the software for analysis. In general, 110 initial codes emerged in this section and eventually became three sub-themes and a main theme. This analysis was managed in MAXQDA software version 10.

### Rigor

To ensure the rigor of findings, the researchers increased their credibility by devoting sufficient time to evaluating the raw interview data and following the step-by-step analysis process. They also increased the data richness by establishing a good communication with the participants and gaining their trust. The findings were offered to a number of participants for review to see whether they are compatible with the participants’ experiences. Also, two experts were asked to assess the accuracy of analysis and extracted themes, and their perspectives were applied. All steps of the study process were reported to increase an audit trail. Last of all, a precise description of the participants was recorded. This could help to review the transferability of findings to other situations and patients [20].

## Results

In this study, half of the participants with SCI had thoracic injury. Most of them lived in Tehran and were unemployed. Participants’ profiles are presented in the Table 1.

**Table 1:** Demographic characteristics of the studied participants

Variables	Individuals with SCI (N)	Family caregivers (N)	Median (Interquartile range)
Age range (year)	24–63		36 (29.5–43.5) → SCI people
			52 (42.5–57.5) → Family caregivers
Sex			
Woman	3	8	
Man	14	5	
Education			
Primary school	1	5	
Secondary school	10	8	
College	6	0	
Marital status			
Single	7	1	
Married	7	11	
Divorced	3	1	
Cause of Injury			
Car accident		14	
Falling		2	
Hit the bullet		1	
Job status			
Student	0	1	
Worker	0	1	
Self-employed	1	2	
Employee	2	7	
Unemployed	14	2	
Range of length of time since SCI (month)	12–192		36 (24–108)
American SCI Association Impairment Scale			
A	7		
B	4		
C	5		
D	1		
Level of injury			
C1-C7	5		
T1-T12	10		
L1-L5	2		
Type of relationship			
Wife		3	
Father		2	
Mother		6	
Daughter		1	
Brother		1	

In general, 110 initial codes which were the result of semi-structured interviews and observations emerged in this section of the study. Interview questions are presented in the Table 2. Eventually, data analysis revealed three sub-themes: 1) physical rehabilitation by various methods, 2) tendency towards the use of alternative medical methods, and 3) making effort for self-reliance and one main theme (towards overcoming physical disability).

**Table 2:** The interview questions

For people with SCI:	
1) From morning until night, what do you do to increase physical ability?	
2) What facilities for physical independence/recovery are available for you to help you?	
3) How do you help your physical recovery/independence? Please give an example.	
For family caregivers:	
1) Please explain how you take care of your SCI family member in one regular day?	
2) How do you handle the care? Please give an example.	
3) How can you help to person with SCI in physical rehabilitation?	

### Towards overcoming physical disability

All participants with SCI had been trying to overcome physical disability. Also, their family caregivers had been trying to help them return to normal life; as well as, their physicians and paramedics had tried to help them in this issue with ordering physical rehabilitation activities and activities daily living education. Therefore, most of our participants with SCI had undertaken physical rehabilitation with the help of their families. Other efforts such as “using alternative medical methods” often had been tested by the affected person and their families. However, most of them had been spending time and money ineffectively. Another effort was self-reliance, which was made by the participants through the long process. Lack of necessary training at home to achieve self-reliance had made the process longer. In Table 3 themes and sub-themes have been written.

**Table 3:** Theme & Sub-theme extracted of codes obtained from experiences of spinal cord injury patients and their families

Major theme	Sub-theme	Sub -subtheme	Sample code
Towards overcoming physical disability	Physical rehabilitation by various methods	Maintaining organ function with physical exercises	Attention and maintaining of organs above lesion level
		Establishing an appropriate living environment as much as possible	Making a slope in the yard, transferring to townhouse, buying bath seat/assisted machine/three-wheel motor
		Using special rehabilitation services	Physiotherapy, Occupational therapy
		Using physical aids’ equipment	Cane, walker, wheelchair, brace, AFO, KAFO
	Tendency towards the use of alternative medical methods	Testing the homeopathy	Strengthen the body with homeopathic medicines
		Testing the acupuncture	Traveling to different cities for acupuncture, incontinence control with acupuncture
		Testing the traditional medicine	Putting honey on bedsore, herbal medicines, blood giving
		Testing the energy therapy	Energy therapy to reduce pain, energy therapy to overcome disability
		Testing the hydrotherapy	Water therapy services, swimming pools
		Testing the stem cell therapy	Cell infusion to restore a sense of intrathecal injection
	Making efforts for self-reliance	Innovation of family in manufacturing a rehabilitation tool	Customized baby walker in big size, ceiling ropes
		Trying to become independent in personal activities	Patient efforts: Trying to toilet and catheterization without help, eating and bathing alone without training by health care team
			Family efforts: not helping the SCI patient for moving from wheelchair to the bed, putting the items close to the SCI patient

#### Physical rehabilitation by various methods

Most of the participants had been referred to an occupational therapy and had been given simple mechanical tools such as hand and foot pedals for exercises at home. This sub-theme was extracted by four other sub-themes: 1) maintaining organs function with physical exercises, 2) establishing an appropriate living environment as much as possible, 3) using special rehabilitation services, and 4) using physical aids and equipment. Each of these sub-themes is described below:

#### Maintaining organ function with physical exercises

The efforts made by the physical therapists, occupational therapists, family caregivers and individuals with SCI in this subtheme include: A) increasing the motion range and preventing articular deformities with active exercises (by the individuals with SCI) and inactive exercises (by occupational therapist), B) prescribing appropriate means of fixing (such as splints), and C) putting the injured person in correct situation that would increase muscle strength. The primary data categories that created this sub-theme were:

#### Increasing the disabled organs’ strength above the lesion level

Most participants considered exercises as essential factor in increasing organs’ function above the lesion level.

“Since then, I do the upper body exercises. Now, I (the disabled) need strong hands and arms to move easily as I am a little overweight.” (Paraplegic man, 32-year-old)

#### Maintaining organ function below the lesion level

Almost all participants did the below lesion level exercises to increase articular movement range and this was mostly noted in those who had been injured for a long time.

“At the beginning, there were some signs days before the injury when I was healthy. But they began to fade away as the time passed by. The more you improve those remaining abilities; there is more possibility to keep them. For example, I saw a patient with the same injuries, but he did not do anything except for sitting on the wheelchair; so his foot became the size of my hands; but I did a lot of exercises with the help of the therapist and my mother.”

(Tetraplegia man, 32-year-old)

#### Establishing an appropriate living environment as much as possible

Most participants only carried out part of what is needed to adjust the home for a person with SCI. However, others were not able to change the home because of being a tenant. So, after living in difficult circumstances for some time, they had to move to a townhouse which is more comfortable for transferring.

“We used to live in a duplex home, so I made a stair rail for her to come downstairs with; but since I have to put her on and off the chair each time we decided to rent a townhouse for convenience.” (Husband of a paraplegia woman, 62-year-old)

#### Using special rehabilitation services

Almost all participants had experienced physiotherapy and occupational therapies, even for a short term. They had noted that, these approaches are effective and essential.

“I come here for occupational therapy three times a week, and I also went to a rehabilitation institute; which was offering balance exercises”. (Paraplegic woman, 39-year-old)

Some individual with SCI prepared some available tools in the occupational therapy to use them whenever required.

“I bought foot pedals and I thought it would help him to exercise his feet, now we are going to buy hand pedals for home exercises.” (Daughter of a tetraplegia man, 25-year-old)

#### Using physical aids’ equipment

Equipment such as canes, walkers, wheelchairs, braces, and Ankle Foot Orthotic (AFO) would reduce the dependency of individuals with SCI and improve their functional independence. Almost all of our participants used some sort of aids and equipment depending on their economic status.

“AFO is a plastic tool for the legs to take a few steps, however, I should hold on to something else. But I am glad that I can still stand on my own foot.” (Paraplegic man, 28-year-old)

Some of the studied participants with SCI had bought the typical physical auxiliary equipment, but others had managed to make their own electric hoists or wheelchairs at home.

“Since it is too difficult for him to move, I bought these electric lifting hoists; so I put him at the electric lift to wear his pants or sit on his wheelchair”. (Mother of a tetraplegia man, 52-year-old)

#### Tendency towards the use of alternative medical methods

Some of studied individuals with SCI used alternative or complementary medicine based on the advice of relatives or friends to improve excretion or reduce physical pain. They were looking for recovery. Furthermore, some of them did not do anything else and did not even have job. They had experienced homeopathy, acupuncture, traditional Persian medicine, energy therapy, water therapy and stem cell therapy.

#### Testing the homeopathy

Participants used plants and minerals instead of chemical drug witch they called homeopathy. They believe these nature materials stimulate the healing process. In this regard, one of the participants with SCI stated:

“A friend who was suffering from headache was satisfied with homeopathy; I asked her about the technique and she said: "this method helps the body to begin repairing itself, so people can reduce drug consumption. I went there for pain and incontinence and now after a few months, I feel a little better.” (Paraplegia woman, 47-year-old)

#### Testing the acupuncture

Most of the individuals with SCI were dissatisfy of acupuncture and called it “ineffective” or “waste of time and money”.

“I went to Zanjan city for acupuncture and it was not useful. I wasted my time and money. I’m not doing anything else and looking for recovery” (Tetraplegia man, 31-year-old)

#### Testing the traditional Persian medicine

In Iranian traditional medicine everybody has a definite temperament which is determinant to construct all physical or mental characteristics. Some individuals with SCI had used traditional Persian medicine to improve their digestive and motion status.

“Sure! The traditional medicine was quite effective; I was crawling like little kids, but now I can standup. The traditional bloodletting also makes me feel better every time.” (Paraplegia man, 52-year-old)

#### Testing the energy therapy

Few participants with SCI were undergoing energy therapy to relieve pain and severe spasms. Participants cited that energy therapy means the touch by energy therapist for health and peace.

They stated that, it had good short-term effects.

“I have a friend who introduced me for energy therapy. I went there and energy therapist touch me, during the energy therapy, my pain stopped for few minutes.” (Paraplegia man, 37-year-old)

#### Testing the hydrotherapy

Most of participants with SCI had used water therapy to increase their physical strength according to physicians’ suggestion or advice of friends.

“My doctor told me to go to the pool, and now I think it is so effective as it is softening my joints.” (Paraplegia man, 40-year-old)

#### Testing the stem cell therapy

Most participants wanted to try the stem cell therapy (special cells infusion) to restore the sense and movement of the disabled limbs. Some of them satisfied with doing that and few of them were dissatisfied and rest of them didn’t know about the result and they didn’t have comment.

“I have heard, this place offers cell therapy here, so I came to talk to the doctor about my son. This is his second injection and it is relatively expensive and I am waiting for the results.” (Mother of a paraplegia man, 51-year-old)

#### Making effort for self-reliance

Most participants tried to change from physical dependence to independence as much as possible. This sub-theme had two sub- sub- themes which were “innovation of family in manufacturing a rehabilitation tool” and “Trying to become independent in personal activities.”

#### Innovation of family in manufacturing a rehabilitation tool

Few individual with SCI had done innovations with help of their families to improve their physical conditions. They claimed that there was no equipment available that they needed for physical rehabilitation.

“I saw something like a baby walker and since I was not able to stand up, I decided to make a large baby walker in big size for myself. I told my family about it and they helped me so it was made by a friend blacksmith. Now, I use the big-sized baby walker and I am satisfied with it.” (Paraplegia man, 28-year-old)

#### Trying to become independent in personal activities

Both the injured people and their families tried to make the disabled person independent in doing his/her personal activities. Sometimes the person decided to become independent and at other times the family did not help the person in personal tasks on purpose to make the disabled person to become independent. All participants stated that they had no training on this issue.

“My brother helped me a lot to become independent. He prevented others to feed me so I had to learn how to eat by myself.” (Tetraplegia man, 36-year-old)

## Discussion

Our findings showed that, people with SCI and family member caregivers have different ways to improve disabled people’s physical functions. These methods include physical rehabilitation, using alternative medical methods, and making efforts for self-reliance.

Physical rehabilitation by various methods was a theme which was obtained in this research. This theme had several sub-themes, including maintaining organs’ function with physical exercise, establishing an appropriate living environment as much as possible, and using special rehabilitation services and physical aids. In this regard, Hicks et al. (2011) stated that, loss of fitness and independence has a significant relationship with the lack of physical activity. They stated that people with SCI perform exercise to achieve physical ability in long-term [21]. According to Kehn and Kroll (2009), people with SCI have a great passion for physical activities, although they encounter obstacles such as lack of access to equipment, finance to use the equipment, or fear of injury [13].

In our study, most participants had tried to have the maximum assisted environment. Vissers et al. (2008) showed that many SCI persons have movement problems at home due to the lack of assistance, and limited physical activities, although they do their best to obtain the required assistance [22].

Most of our participants had been testing alternative medical treatment methods including the homeopathy, energy therapy, acupuncture, and traditional Persian medicine, water therapy, and stem cell therapy to improve physical functions and improve their restrictions such as urinary and fecal incontinence, pain, and spasms.

Some studies have shown that disabled people use stem cell therapy to improve their motor function [23]. However some of treatments with stem cell are waste of money, and create risk to health [24]. Teng et al. (2006) showed patient’s physical activity is a multithreaded process that includes the cell therapy [25]. Another study by Van Buyten et al. (2001) found that complementary therapies such as acupuncture, massage, and percutaneous electrical therapies may reduce the pain in people with SCI [26]. In a study on the effect of water therapy on spasms, Kesiktas et al. (2004) showed that water therapy reduces spasms in disabled people. This method is always used as a complementary medicine [27].

Making efforts for self-reliance in people with SCI and efforts of family members for their independence was another theme found in this research. They intended to reinforce the physical independence with innovation in manufacturing rehabilitation tools and not helping the disabled person. The findings of Kerstin et al. (2006) study were similar to the result of present study in this regard. They mentioned some of the behavioral strategies that a person uses to improve physical activity after SCI [28].

One of the limitations of this study was that, the participants had a diverse range of SCI. Since different type of SCI might be accompanied by different challenges and experiences, some of the findings might not be generalized to a larger population.

Another limitation in this study was that, we did not interview individuals with SCI living in remote areas such as villages that did not have access to rehabilitation services.

## Conclusion

Overcoming physical disability in people with SCI is a difficult and long term process. In this study, most of participants used special rehabilitation services as an effective and essential approach offered by the health care team. Some individuals with SCI with help of their family had invented simple rehabilitation equipment for help to their physical rehabilitation.

Individuals with SCI had also tested alternative medical methods based on advice of relatives and friends and they often had spent a lot of time and money. Even they did not do anything for a long time.

Therefore, they need formal activities of daily living rehabilitation in an institutional setting in Iran following SCI in acute phase as well as social support to overcome physical disability as soon as possible and return to society and normal life.

Our findings can help the health policymaker in developing countries to provide more community- based services with high quality to people with SCI and their families. Also, policymakers can re-regulate the healthcare guidelines to facilitate the integrated healthcare system for the people with SCI. It seems that home care training programs offered by nurses and occupational/physiotherapists and having a family physician can be used as effective solutions. Further research is suggested to investigate the barriers to achieving physical empowerment in people with SCI in Iran.

## Data Availability

The datasets generated and/or analyzed during the current study are not publicly available due to confidentiality and privacy of the participants but are available from the corresponding author on reasonable request.
